# The Triglyceride/HDL Ratio as a Surrogate Biomarker for Insulin Resistance

**DOI:** 10.3390/biomedicines12071493

**Published:** 2024-07-05

**Authors:** Petru Baneu, Cristina Văcărescu, Simona-Ruxanda Drăgan, Liviu Cirin, Alexandra-Iulia Lazăr-Höcher, Andreea Cozgarea, Adelina-Andreea Faur-Grigori, Simina Crișan, Dan Gaiță, Constantin-Tudor Luca, Dragoș Cozma

**Affiliations:** 1 Doctoral School, “Victor Babes” University of Medicine and Pharmacy, 300041 Timisoara, Romanialiviu.cirin@umft.ro (L.C.); alexandra.hocher@umft.ro (A.-I.L.-H.); andreea.cozgarea@umft.ro (A.C.); 2 Department of Cardiology, “Victor Babes” University of Medicine and Pharmacy, 2 Eftimie Murgu Square, 300041 Timisoara, Romania; simona.dragan@umft.ro (S.-R.D.); simina.crisan@umft.ro (S.C.); dan.gaita@umft.ro (D.G.); constantin.luca@umft.ro (C.-T.L.); dragos.cozma@umft.ro (D.C.); 3 Institute of Cardiovascular Diseases Timisoara, 13A Gheorghe Adam Street, 300310 Timisoara, Romania; andreeaadelinafaur@yahoo.com; 4 Research Center of the Institute of Cardiovascular Diseases Timisoara, 13A Gheorghe Adam Street, 300310 Timisoara, Romania; 5 County Clinical Emergency Hospital of Sibiu, 550245 Sibiu, Romania

**Keywords:** TG/HDL ratio, insulin resistance, surrogate biomarker, metabolic syndrome

## Abstract

Given the widespread occurrence of insulin resistance, a key factor in metabolic syndrome and a distinct condition altogether, there is a clear need for effective, surrogate markers. The triglyceride-to-high-density lipoprotein (TG/HDL) ratio stands out as a viable option, indicative of changes in lipid metabolism associated with insulin resistance, offering a cost-effective and straightforward alternative to traditional, more complex biomarkers. This review, in line with PRISMA guidelines, assesses the TG/HDL ratio’s potential as an indirect indicator of insulin resistance. Analysing 32 studies over 20 years, involving 49,782 participants of diverse ethnic backgrounds, including adults and children, this review primarily uses a cross-sectional analysis with the Homeostatic Model Assessment for Insulin Resistance (HOMA-IR) to gauge insulin resistance. It reveals the TG/HDL ratio’s varied predictive power across ethnicities and sexes, with specific thresholds providing greater accuracy for Caucasians, Asians, and Hispanics over African Americans and for men over women. Valid across different weights and ages, for adults and children, it suggests average cutoffs of 2.53 for women and 2.8 for men. The analysis supports the TG/HDL ratio as a simple, accessible marker for insulin resistance, though it advises further research on tailored cutoffs reflecting ethnic and gender differences.

## 1. Introduction

Insulin resistance (IR) is a widespread health concern worldwide, with precise global prevalence figures being challenging to pinpoint due to variations in diagnostic criteria and population studies. It is most associated with obesity and is a core component of metabolic syndrome (MS) [1,2]. Even though this association is completely justified, IR is starting to take shape as a separate entity. Recently, the TOFI (thin-outside-fat-inside) phenotype has been used to describe lean individuals with a disproportionate amount of visceral fat, who are insulin-resistant [1]. On the other hand, the MHO phenotype (metabolically healthy obese) represents a unique subset of euglycemic individuals who bear significant amounts of subcutaneous adipose tissue but display favorable lipid profiles, an absence of systemic inflammation, and normal liver function [2]. Therefore, IR, MS, and obesity are evidently intertwined and interdependent but not superposable. Thus, search on the dedicated biomarkers of IR is more prevalent than ever.

Despite this obvious and urgent necessity, quantifying IR has proven to be a significant challenge in clinical and research settings due to the complexity of its underlying mechanisms and the lack of a universally accepted measurement standard [1,2,3]. Traditional direct methods, such as the hyperinsulinemic–euglycemic clamp technique, are often deemed impractical for routine clinical use, owing to their invasiveness, technical complexity, and resource-intensive nature. Consequently, the medical community has sought alternative, indirect biomarkers that could serve as surrogates for assessing insulin sensitivity.

HOMA-IR (Homeostatic Model Assessment for Insulin Resistance) and FPI (Fasting Plasma Insulin) are both commonly used as surrogate markers for assessing IR with a strong predictive power, high specificity, and sensitivity. Nonetheless, even though they are more feasible than the hyperinsulinemic–euglycemic clamp technique, they are still cumbersome in daily practice [2,3,4,5]. Therefore, the search for simpler biomarkers to be integrated into a standard examination continues unabated.

The triglyceride-to-high-density lipoprotein (TG/HDL) ratio has been proposed as an indirect marker of IR. This is because the metabolic processes that result in IR also lead to changes in lipid metabolism, which are reflected in the levels of serum triglycerides and HDL cholesterol [2]. A brief dive into the pathophysiology of IR reveals that one of the physiological functions of insulin is to inhibit the release of free fatty acids from adipose tissue and promote the storage of triglycerides in adipocytes [4]. However, in IR, this regulatory mechanism is impaired. Firstly, an increased rate of unchecked lipolysis leads to an accelerated release of free fatty acids (FFAs) into the bloodstream [4]. Consequently, the liver, inundated with excess FFAs, increases triglyceride production, packaging them into very low-density lipoprotein (VLDL) particles, resulting in hypertriglyceridemia. On the other hand, IR also augments the catabolism of HDL particles, partly due to the heightened activity of hepatic lipase, an enzyme that hydrolyzes HDL triglycerides and phospholipids, leading to smaller and less stable HDL particles that are more rapidly cleared from circulation [4,6]. Additionally, IR impairs the synthesis of apolipoprotein A-I (ApoA-I), a primary structural protein of HDL. Finally, IR alters the structural characteristics of HDL, making it more prone to oxidative stress, negatively affecting its protective properties [3,4,6]. The resulting lipid profile, characterized by elevated triglycerides and reduced HDL cholesterol, can be succinctly represented by the TG/HDL ratio.

The unequivocal simplicity and accessibility of these two biomarkers have encouraged the pursuit of defining the exact predictive capability, limitations, and peculiarities of this ratio over the last two decades.

This systematic review has as the main objective of assessing the feasibility of utilizing the TG/HDL ratio as a surrogate marker for IR.

## 2. Materials and Methods

The methodology for selecting studies in this review adhered to the PRISMA (Preferred Reporting Items for Systematic Reviews and Meta-Analyses) guidelines, being registered on the PROSEPRO platform, with the following code: CRD42024545597. The process entailed an extensive bibliographic examination using databases such as the PubMed database and Google Scholar, using “TG/HDL ratio” and “insulin resistance” as keywords.

### 2.1. Eligibility Criteria 

The inclusion criteria were represented by prospective or retrospective observational studies that included nondiabetic adults or children and that were aimed at quantifying the predictive value the TG/HDL ratio as a surrogate marker of IR. The IR was measured utilizing the gold standard test—the hyperinsulinemic–euglycemic clamp technique or other consecrated surrogate markers for IR.

### 2.2. Electronic Search and Study Selection

The determination of the most relevant articles was based on a thorough evaluation of their titles, the content of their abstracts, and a preliminary review of the complete manuscripts. Articles that were not in English, publications that were only available as abstracts, and redundant entries were systematically excluded from this review.

A total of 32 scientific papers were selected and included in this review. The studies are listed in Table 1 and Table 2, along with the test of IR utilized, the TG/HDL ratio for women and men, respectively, the BMI (Body Mass Index) of the participants, and the number of participants. Tabel 1 comprises the studies that enlisted less than 1000 participants, whereas Tabel 2 displays those with studies with more than 1000 participants.

**Figure 1:**
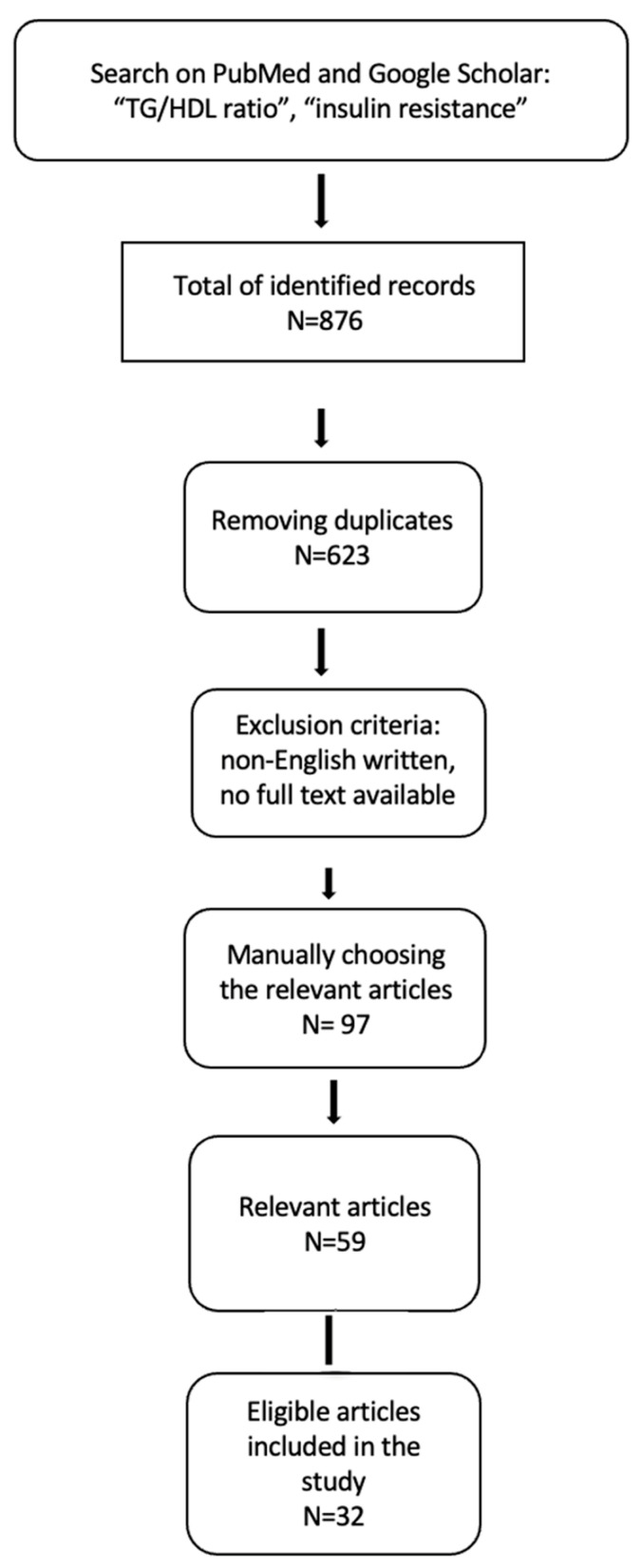
Search strategy employed in this review.

The PRISMA diagram below illustrates the search strategy employed, along with the filters that were applied (Figure 1). 

## 3. Results

### 3.1. Overview

#### 3.1.1. Participant Numbers and Demographics

Over the course of two decades, spanning from 2003 to 2023, our review encompassed 32 studies involving a cumulative total of 49,782 participants. The scope of these studies varied considerably, ranging from a minimal cohort of 61 children to a substantial assembly of 10,132 adults. Within this spectrum, 7 studies specifically investigated pediatric subpopulations, whereas 25 studies primarily focused on adult demographics. Distinct studies targeted gender-specific populations, with three dedicated to female participants, one to male participants, and the remainder incorporating a gender-inclusive approach. 

#### 3.1.2. Ethnic and Racial Diversity

The studies examined a wide array of subpopulations including, but not limited to, Caucasians, African Americans, Asians, and Hispanics.

#### 3.1.3. Classification of Study Types

Predominantly, the research methodologies employed were of a cross-sectional nature, with 24 such studies conducted. Additional methodological approaches included clinical transversal observational studies and retrospective cohorts.

#### 3.1.4. Statistical Methods

The analytical techniques employed across the studies were diverse. Notably, the Receiver Operating Characteristic (ROC) curve analysis was utilized in 17 studies with an Area Under Curve (AUC) exceeding 0.7, which signified a reasonable predictive capacity. The odds ratio (OR) analysis was another prevalent method, which was applied in 12 studies.

The Homeostatic Model Assessment for Insulin Resistance (HOMA-IR) emerged as the predominant metric for assessing IR in 28 studies. It assesses IR and quantifies the beta-cell function by integrating fasting blood glucose and fasting insulin levels [39]. Its simplicity and non-invasiveness are the reasons for its widespread acceptance and use in most scientific scenarios [39]. Fasting serum insulin (FSI) is another marker that measures the concentration of insulin in the blood after an overnight fast. Despite being straightforward, it does not provide a complete picture of insulin sensitivity, which generally restricts its applicability [40,41]. The third test found was the quantitative insulin sensitivity check index (QUICKI), which is derived from fasting blood glucose and fasting insulin levels and is considered both reliable and simple [42]. The steady-state plasma glucose concentration during an insulin suppression test is a more direct and invasive method, providing an accurate assessment of IR, but it is less commonly used due to its complexity, even in studies [43,44]. The frequently sampled intravenous glucose tolerance test (FSIVGTT) involves administering a glucose injection, followed by frequent blood sampling, to measure glucose and insulin levels over time, with the data then analyzed using the minimal model analysis to estimate insulin sensitivity. This is probably the most time-consuming and labor-intensive method [45,46]. Lastly, the hyperinsulinemic–euglycemic clamp technique represents the gold standard for measuring insulin sensitivity and assessing insulin resistance. By inserting concomitant intravenous lines, insulin and glucose are infused, with the measurement of the glucose infusion rate (GIR), which reflects the amount of glucose necessary to maintain euglycemia under hyperinsulinemic conditions. This rate is directly proportional to insulin sensitivity. This method is also labor-intensive, costly, and requires significant expertise [47,48].

#### 3.1.5. Body Mass Index (BMI) of Participants

The studies exhibited a wide range of participant BMIs, from those classified as overweight or obese (13) to individuals of normal weight (4). Most of the studies did not have the BMI as an exclusion criterion, but with no exception, this parameter was noted.

### 3.2. Comorbidities and Chronic Treatment

In the total of 32 studies included in our review, no patient was on medications that could interfere with the lipid parameters. Moreover, the participants were free of known major diseases, such as heart disease, peripheral vascular disease, liver disease, thyroid and other endocrine diseases, or neoplasms. In only one of the studies, 20% of the subjects were reported to have hypertension [9].

### 3.3. Ethnicity and Gender

In 2003, McLaughlin et al. were the first to mention a correlation between the TG/HDL ratio and IR in overweight nondiabetic patients. They utilized a cutoff of 3.0 for the TG/HDL ratio, finding a AUC-ROC of 0.781 (CI 95%). Despite this encouraging percentage, this study had several limitations that have been briefly noted in the literature: the patient cohort consisted mostly of Caucasians, with only 1% African Americans, and did not differentiate based on gender, neglecting normal-weight subjects, who can also be affected by IR [7]. Two years later, in 2005, the same team further explored this issue by conducting another study involving twice as many healthy, nondiabetic individuals [8]. Gender, BMI, and age were reported to bear no statistical significance, and the cutoff utilized this time was 3.5, which has been described as indicative of IR. During the same year, Summer et al. published an article focusing on the utility of the TG/HDL ratio in predicting IR in a population of African Americans. The number of patients was relatively small—125—but the results were unequivocal: the AUC-ROC for a TG/HDL of >3 was just 0.56 ± 0.05 (CI 95%) [9].

The Asian subpopulations were examined in the years that followed, with 7 studies focusing on South Korean, Japanese, Chinese, Malaysian, and Taiwanese populations, totaling 18,158 adults and 271 children [14,17,20,21,26,33,34,49]. The TG/HDL ratio was generally portraited as a capable surrogate marker of IR, with several observations that deserve to be noted: Chiang et al. in 2011 found that additional clinical factors such as gender, waist circumferences, and ALT levels can augment the diagnostic accuracy, while Kang et al. and Kim et al., one year later, noted that the TG/HDL ratio is linearly associated with IR independently of waist circumference in both genders [14,26,33,34]. Zhou et al. and Iwani et al. also advocated for the predictive value of the TG/HDL ratio for IR, without insisting on differences based on gender or anthropometric elements [20,21]. In contrast, He et al., in 2014, published an article with Chinese adult participants and found that the TG/HDL ratio was adequate for discriminating IR mainly in non-obese women [17].

The Hispanic population received separate attention in research studies. Either with varied ethnic subgroups or by solely enlisting Hispanic participants, several papers have conveyed that the TG/HDL ratio is a useful tool for predicting IR [15,22,23,29,35,36]. In 2008, Li et al. addressed the question of ethnicity, finding no relevant difference in the OR of the three separate subpopulations of their study, non-Hispanic whites, non-Hispanic blacks, and Mexican Americans, respectively, with the TG/HDL ratio of >3.5 showing a considerable predictive value for IR. After adjustments for potential confounding effects, the prevalence ratio of hyperinsulinemia was 2.16 (95% confidence interval [CI], 1.74 to 2.08) when using a single cutoff point of 3.5 and 2.23 (95% CI, 1.83 to 2.72) when using race/ethnicity-specific cutoff points of 3.0 for non-Hispanic whites and Mexican Americans and 2.0 for non-Hispanic blacks for the TG/HDL-C ratio. The AUC-ROC of the TG/HDL-C ratio for predicting hyperinsulinemia was 0.77 (95% CI, 0.74 to 0.79); 0.75 (95% CI, 0.69 to 0.77); and 0.74 (95% CI, 0.69 to 0.76) for non-Hispanic whites, non-Hispanic blacks, and Mexican Americans, respectively [29]. Gonzalez et al., Salazar et al., and Murguia-Romero et al. conducted studies focusing only on the Hispanic subpopulation, demonstrating the predictive power of the TG/HDL ratio and proposing various cutoff values, either as one common number regardless of gender or with variations based on it [15,35,36]. In 2018, Borrayo et al. focused on a subpopulation of Hispanic women, reporting strong predictive values for a TG/HDL ratio of >3 both in non-obese and obese subjects with an OR of 3.27 (95% CI) and 4.7 (95% CI), respectively [22]. In 2019, Pantoja-Torres et al. highlighted that the TG/HDL ratio could be used for assessing IR in euthyroid normal-weight adults [23].

### 3.4. Gender Particularities

Gender-specific differences have also emerged as a significant factor to be taken into consideration. In 2007, Karelis et al. analyzed 131 overweight or obese nondiabetic women, concluding that the TG/HDL ratio alone could serve as a reliable tool in identifying women with IR, mainly in the obese, post-menopausal subpopulations [11]. Eight years later, Maturu et al. stated that the TG/HDL-C ratio is a poor predictor of IR in African American women [19]. In contrast, in 2018, Borrayo et al. focused on a subpopulation of Hispanic women, reporting strong predictive values for a TG/HDL ratio of >3 both in non-obese and obese subjects, with odds ratios of 3.27 (95% CI) and 4.7 (95% CI), respectively [22]. He et al. evaluated a subpopulation of Chinese subjects segregated by gender and BMI, reporting a predictive TG/HDL ratio only for the group of non-obese women with an AUC-ROC of 0.718 (95% CI) [17].

### 3.5. Children Particularities

The pediatric population has been examined separately. The 7 studies that were included in our review spanned over a period of 13 years and included children between 5 and 18 years of age, with various ethnic backgrounds (Caucasian, Asian, Hispanic, African American, and Egyptian) [12,18,21,24,25,27,32]. Atotal of 6 of these studies concluded that the TG/HDL ratio has a significative predictive value for IR in pediatric populations, regardless of gender, age, or ethnicity, with a reported OR of 2.58 by Hirschler et al. and 2.47 by Iwani et al. [18,21]. The AUC-ROC found by Giannini et al. was 0.706 [32]. Behiry et al. stated that a TG/HDL ratio of ≥1.36 had 85.7% sensitivity and 66.7% specificity, while Garcia et al. found an AUC-ROC of 0.729 for a TG/HDL ratio above 2.22 [24,25]. Quijada et al.’s work (2008) was the only study that depicted a lack of correlation of the TG/GHDL ratio with insulin sensitivity indexes like HOMA or QUICKI but demonstrated that a significant proportion of obese children, those with hypertension and those with MS, exhibited a TG/HDL-C ratio of 3.5 or higher [12].

### 3.6. TG/HDL Ratio Cutoff Values

One important aspect in clinical practice is represented by TG/HDL ratio cutoff values. In 50% of the studies, specific cutoff values were proposed by the authors, with the rest having used the TG/HDL ratio as a continuous variable. In the case where exact numbers were attributed to the ratio, it was either a general one for the entire subpopulation, or several cutoffs were recommended in functions of race or gender. The lowest ratio was 1.36 for both genders in children, as proposed by Behiry et al. in 2019 [24]. The highest one was 3,5 for both genders, as was postulated in 3 separate studies between 2005 and 2008 [8,10,12]. The median cutoff value for women was 2.53, whereas the median one for men was 2.8. In 2008, Li et al. addressed the question of ethnicity, finding no relevant difference in the OR of the 3 separate subpopulations of their study, non-Hispanic whites, non-Hispanic blacks, and Mexican Americans, respectively, with the TG/HDL ratio, when using ethnicity-specific cutoff points, of 3.0 for Caucasians and Mexican Americans and 2.0 for African Americans [29].

### 3.7. The Triad TG/HDL, IR, and BMI

In most of the studies, when a statistical analysis was used to filter the altering effects of the BMI as a confounding factor for the predictability of the TG/HDL ratio for IR, an independent correlation was indeed reported. On the other hand, Gong et al., in 2021, described an inverse correlation between the TG/HDL ratio and the BMI, the strongest association being in subjects with a BMI of 18.5–24 [38]. Pantoja-Torres et al. concentrated only on normal weight healthy adults in demonstrating the predictive value of the TG/HDL ratio [23]. Yeh et al., in 2019, reported the opposite: the TG/HDL ratio was higher as the BMI value increased [26]. Borrayo et al., in 2018, described the TG/HDL ratio as a capable predictive biomarker for IR in both overweight/obese and normal weight women [22]. He at al., in 2014, concluded that the discriminatory power of the TG/HDL ratio for IR differs by the gender and obesity index in the normo-glycemic Chinese population, and the TG/HDL ratio could discriminate IR in non-obese and normoglycemic women [17].

## 4. Discussion

### 4.1. TG/HDL Ratio and Ethnicity

It is a known fact that differences between the lipid profiles in African American populations in contrast with the Caucasian population exist. Genetic, dietary, lifestyle, socioeconomic, and environmental factors are implied [50]. Moreover, research indicates that the activity of lipoprotein lipase (LPL) tends to be stronger in African Americans compared to Caucasians [51]. LPL is a critical enzyme in lipid metabolism, playing a significant role in the hydrolysis of triglycerides in lipoproteins into free fatty acids and glycerol, thereby facilitating the clearance of triglycerides from the bloodstream. In addition, African Americans often have higher average HDL cholesterol levels compared to Caucasians [50]. This difference is observed across various age groups and genders. Several studies within African American populations by Summer et al. and Kim-Dormer et al. have confirmed the disparities in the ratio’s effectiveness, indicating a weaker association in contrast to Caucasian cohorts [13,31]. This disparity was further explored by Bovet et al. and others, who expanded the ethnic spectrum, incorporating Africans, mixed ethnic groups, and non-Africans, thereby reinforcing the concept that metabolic markers cannot be universally applied without considering ethnic-specific thresholds [10,31]. Even so, not all the studies including African Americans point towards the idea that the TG/HDL ratio is an inappropriate surrogate marker for IR in this subpopulation. Large studies, such as the one published by Li et al. comprising 2652 adults, reported a favorable predictive value across all evaluated ethnic groups (Caucasians, Hispanics, and African Americans) [29]. Moreover, the largest one, enlisting more than 10,000 subjects of various ethnic origins, including more than 20% African Americans, concluded that the TG/HDL ratio is positively associated with IR in a nonlinear interaction pattern. In regards to the discrepancies noted among the African American populations in comparison to Caucasians, Asians, or Hispanics, lower cutoffs of 2.5 or even 2.0 appear to be more appropriate [38].

### 4.2. TG/HDL Ratio and Gender

Another possible altering factor is gender. Women, particularly in the premenopausal age, are known to have more favorable lipid profiles, which can evidently decrease the sensitivity and specificity of the TG/HDL ratio. If the women are of African American ethnicity, the effect may become cumulative, necessitating adjusted cutoffs between 1.5 and 2.0. Several means exist to augment the predictive value of the ratio in certain subpopulations. One approach is the addition of anthropometric data or other biomarkers, as demonstrated by Chiang et al., who managed to increase the AUC-ROC from 0.66 to 0.77 by including gender, ALT levels, and waist circumference [13]. Another possibility is to use other indirect biomarkers for IR, such as the triglyceride–glucose index, which also shows promising predictive capabilities [25,52,53].

### 4.3. Overall Predictive Value of TG/HDL Ratio

**Figure 2:**
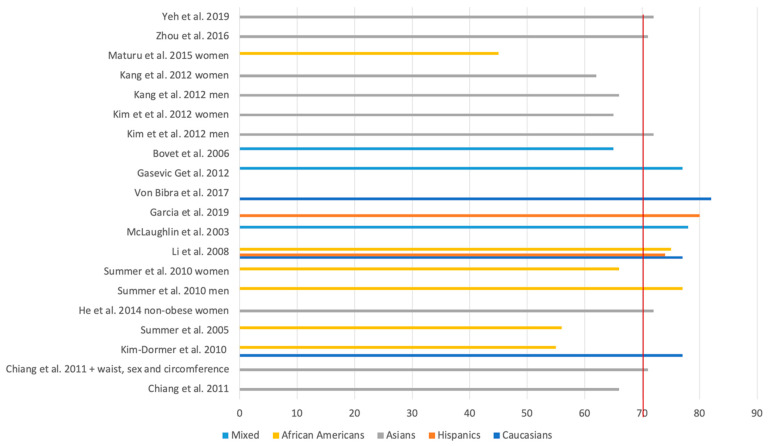
AUC-ROC for the predictive value of the TG/HDL ratio [7,9,10,13,14,16,17,19,20,25,29,31,33,34,37,38].

In half of the articles reviewed, the TG/HDL ratio was reported to present an AUC-ROC above 0.7 (threshold marked by the red line in Figure 2) in cases of mixed subpopulations or Caucasians. Similar conclusions can be drawn regarding the subpopulations of Hispanics and Asians. However, in African Americans, the results were less reassuring. Considering that a value between 0.5–0.7 is labelled as fair discrimination, with good discrimination being seen only in the interval of 0.8–0.9, by analyzing Figure 2, the fact that only 2 of the 17 studies having used this statistical method have ratios equal to or above 0.8 indicates that additional parameters could be useful in order the enhance the predictive value of the TG/HDL ratio.

### 4.4. TG/HDL Ratio Cutoff Values

Probably the most difficult task lies in establishing the proper TG/HDL cutoff, as in half the reviewed studies, no value was even proposed. Considering the mean values obtained from the 17 studies that did discuss this issue, TG/HDL ratios above 2.5 for women and above 2.8 for men seem appropriate for all populations other than African Americans. In the case of the latter, ratios above 1.5 for women and 2 for men, respectively, can be proposed, with further research being needed for a better calibration of the biomarker.

### 4.5. TG/HDL Ratio and Children

Regarding the applicability to children, the studies evaluated in this review strongly conclude towards a valuable predictive power of the TG/HDL ratio as a surrogate biomarker for IR in overweight or obese patients, regardless of age, sex, or ethnicity. A deeper assessment of the capability of this ratio in normal weight children would be appropriate to be able to extend its use.

### 4.6. TG/HDL Ratio and Obesity

As previously mentioned, IR has taken shape as a separate entity, being also able to promote obesity in normal weight subjects and not only act as a repercussion of it. Studies, like the one published by Gong et al. in 2021, validated the predictive value of the TG/HDL ratio for IR in normal weight patients [38,54,55,56,57,58,59,60,61,62,63,64,65].

### 4.7. TG/HDL Ratio and Lipoprotein(a)

Lipoprotein(a) (Lp(a)) is a solid risk factor for cardiovascular diseases, with values that seem to be constant throughout one’s lifetime, which are largely unaffected by lifestyle changes [66,67,68,69,70,71,72]. Despite consistent efforts invested in pharmacological means of lowering it, there is currently no clinically applicable solution. Lately, a peculiar inverse relationship has been observed in a few studies between Lp(a) levels and IR [72]. To the best of our knowledge, the reasons for this are unknown. Several hypotheses have been proposed to explain this. Some suggest that Lp(a) might exert anti-inflammatory or antioxidative effects in certain contexts, potentially mitigating some of the pathways that lead to IR. Others base this situation on genetic factors [73]. As the levels of Lp(a) are primarily genetically determined, this might explain why its relationship with IR can vary across different studies and populations, in either direction. It is worth mentioning that this inverse association is not widely accepted as a general fact, being contradicted by other studies. Moreover, one study by Tian et al. from 2023, which compared two groups of patients with carotid plaque separated into stable and unstable, found that subjects from the latter group displayed significantly higher levels of both Lp(a) and the TG/HDL ratio than those from the former [74]. Therefore, more research is warranted for a better understanding of the pathophysiological role of Lp(a) in the context of IR. Additionally, considering the importance of Lp(a) in the sphere of cardiovascular disease and the strong correlation between IR and cardiovascular risk, further research is needed to assess the role of Lp(a) and the TG/HDL ratio as combined risk predictors in specific situations, as seen in the above-mentioned article.

### 4.8. TG/HDL Ratio and Apolipoprotein B

Apolipoprotein B (ApoB) is a crucial protein component of various lipoproteins, including very low-density lipoproteins (VLDLs), intermediate-density lipoproteins (IDLs), and low-density lipoproteins (LDLs). It plays an essential role in the assembly and secretion of these lipoproteins from the liver into the bloodstream [6]. Elevated ApoB levels, indicative of a high concentration of atherogenic lipoprotein particles, are strongly associated with an increased risk in cardiovascular diseases [74]. In individuals with insulin resistance (IR), the presence of high ApoB levels, combined with other dyslipidemic components such as elevated triglycerides (TGs) and reduced high-density lipoprotein cholesterol (HDL-C), significantly elevates the risk of atherosclerosis and related cardiovascular events [74]. Additionally, incorporating apolipoprotein A-I (ApoA-I), a key component of HDL-C, into the calculation provides the ApoB/ApoA-I ratio, offering insights into the balance between atherogenic and protective HDL particles [6,74]. When considered alongside the TG/HDL-C ratio, which reflects the balance between triglycerides and HDL cholesterol, these two ratios together provide a comprehensive view of lipid abnormalities and cardiovascular risk in subjects with IR.

### 4.9. General Considerations

We would like to emphasize the utmost importance of using standardized pre-test protocols. Patients should undergo a fasting period of 8–12 h before blood sample collection, avoid alcohol and high-fat meals for at least 24 h prior, and discontinue medications that might interfere with lipid levels, as advised by a healthcare provider in accordance with the specific context [75].

Finally, we strongly believe that, apart from the automated determination of lipid levels, the cold flotation test should not be routinely performed in order to prevent procedural variability and provide consistent and reproducible results, using an easily available, simple, and cost-effective method [76].

## 5. Conclusions

Our article represents the first paper to offer an overview of the feasibility of using the TG/HDL ratio as a surrogate biomarker for IR, highlighting both its strengths and limits, in various subpopulations, since its first mentioning in the literature to the present day. As in recent years, IR has been associated with a varied spectrum of pathologies from Alzheimer’s disease and depression to oncologic conditions and a whole plethora of cardiovascular diseases The utility of determining IR becomes of utmost importance and easily expands beyond the domain of metabolic disorders [66,67,68,69,70,71].

The TG/HDL ratio demonstrates significant predictive values for IR, including for non-obese individuals, thereby challenging the conventional association of IR solely with obesity. Moreover, the TG/HDL ratio demonstrates potential as a valuable predictive tool in pediatric populations, indicating its broader applicability beyond adult cohorts.

The mean cutoffs found in the studies were 2.53 for women and 2.8 for men, and this is what we recommend as the limit for raising awareness and conducting further investigations.

Nevertheless, its effectiveness varies across ethnic groups, also presenting gender-specific differences, with specific cutoff values enhancing its precision in certain populations. It demonstrates greater accuracy for Caucasians, Asians, and Hispanics over African Americans and for men over women. Despite its strengths, the AUC-ROC never virtually surpassing the 0.8 upper limit conveys that the predictive capabilities of the TG/HDL ratio leave room for improvement and seems to be more effective when considered alongside other markers and patient-specific factors.

Further research to integrate, in daily practice, cheap and readily available surrogate biomarkers as the TG/HDL ratio is needed to better calibrate their predictive power.

## Figures and Tables

**Table 1:** The 22 studies with less than 1000 participants included in this review.

Study	Test for IR	Cutoff TG/HDL Women	Cutoff TG/HDL Men	Number of Participants	BMI of Participants
McLaughlin et al., 2003 [7]	Steady-state plasma glucose concentration during the insulin suppression test	3	3	258	Overweight
McLaughlin et al., 2005 [8]	Steady-state plasma glucose concentration during the insulin suppression test	3.5	3.5	449	Normal
Summer et al., 2005 [9]	Steady-state plasma glucose concentration during the insulin suppression test	3	3	125	Overweight
Bovet et al., 2006 [10]	HOMA-IR	3.5	3.5	630	Overweight
Karelis et al., 2007 [11]	Hyperinsulinemic–euglycemic clamp and HOMA-IR	-	-	131	Overweight or obese
Quijada et al., 2008 [12]	HOMA-IR and QUICKI (Quantitative insulin sensitivity check index)	3.5	3.5	67	Variable
Kim-Dorner et al., 2009 [13]	HOMA-IR	-	-	149	Variable
Chiang et al., 2011 [14]	HOMA-IR	-	-	812	Overweight or obese
Gonzalez-Chavez et al., 2011 [15]	HOMA-IR	3	3	177	Variable
Gasevic et al., 2012 [16]	HOMA-IR	-	-	784	Variable
He et al., 2014 [17]	HOMA-IR	-	-	533	Normal
Hirschler et al., 2014 [18]	HOMA-IR	-	-	501	Variable
Maturu et al., 2015 [19]	Frequently sampled IV glucose tolerance test (FSIVGTT)	-	-	41	Overweight or obese
Zhou et al., 2016 [20]	HOMA-IR	-	-	379	Variable
Iwani et al., 2016 [21]	HOMA-IR	2.48	2.48	271	Overweight or obese
Borrayo et al., 2018 [22]	HOMA-IR	3	-	253	Variable
Pantoja-Torres et al., 2019 [23]	HOMA-IR	-	-	118	Normal
Behiry et al., 2019 [24]	HOMA-IR	1.36	1.36	90	Overweight or obese
Garcia et al., 2019 [25]	HOMA-IR	2.22	2.22	201	Overweight or obese
Yeh et al., 2019 [26]	HOMA-IR	2.197	2.2	398	Variable
Demiral et al., 2021 [27]	HOMA-IR	-	-	159	Overweight or obese
Sowndarya et al., 2021 [28]	HOMA-IR	-	-	71	Normal

**Table 2:** The 11 studies with more than 1000 participants included in this review.

Study	Test for IR	Cutoff TG/HDL Women	Cutoff TG/HDL Men	Number of Participants	BMI of Participants
Li et al., 2008 [29]	Fasting serum insulin (FSI)	3.2	3.2	2652	Variable
Glueck et al., 2009 [30]	HOMA-IR	-	-	1724	Variable
Summer et al., 2010 [31]	HOMA-IR	-	2.5	1903	Variable
Giannini et al., 2011 [32]	HOMA-IR	2.27	2.27	1452	Overweight or obese
Kim et al., 2012 [33]	HOMA-IR	-	-	7623	Variable
Kang et al., 2012 [34]	HOMA-IR	-	-	8411	Variable
Salazar et al., 2012 [35]	HOMA-IR and fasting serum insulin (FSI)	2.5	3.5	1566	Variable
Murguia-Romero et al., 2013 [36]	HOMA-IR and QUICKI	2.5	3.5	2244	Variable
Von Bibra et al., 2017 [37]	HOMA-IR and QUICKI	1.9	2.8	1932	Variable
Gong et al., 2021 [38]	HOMA-IR	-	-	10,132	Variable

## Data Availability

Data sharing is not applicable.
